# Biological Properties of 12 Newly Isolated *Acinetobacter baumannii*-Specific Bacteriophages

**DOI:** 10.3390/v15010231

**Published:** 2023-01-13

**Authors:** Natalia Bagińska, Marek Adam Harhala, Martyna Cieślik, Filip Orwat, Beata Weber-Dąbrowska, Krystyna Dąbrowska, Andrzej Górski, Ewa Jończyk-Matysiak

**Affiliations:** 1 Bacteriophage Laboratory, Hirszfeld Institute of Immunology and Experimental Therapy, Polish Academy of Sciences, 53-114 Wroclaw, Poland; 2 Laboratory of Phage Molecular Biology, Hirszfeld Institute of Immunology and Experimental Therapy, Polish Academy of Sciences, 53-114 Wroclaw, Poland; 3 Phage Therapy Unit, Hirszfeld Institute of Immunology and Experimental Therapy, Polish Academy of Sciences, 53-114 Wroclaw, Poland; 4 Infant Jesus Hospital, The Medical University of Warsaw, 02-006 Warsaw, Poland

**Keywords:** *Acinetobacter baumannii*, antimicrobial resistance, ESKAPE, gram-negative bacteria, phages

## Abstract

Infections with the opportunistic Gram-negative bacterium *Acinetobacter baumannii* pose a serious threat today, which is aggravated by the growing problem of multi-drug resistance among bacteria, caused by the overuse of antibiotics. Treatment of infections caused by antibiotic-resistant *A. baumannii* strains with the use of phage therapy is not only a promising alternative, but sometimes the only option. Therefore, phages specific for clinical multi-drug resistant *A. baumannii* were searched for in environmental, municipal, and hospital wastewater samples collected from different locations in Poland. The conducted research allowed us to determine the biological properties and morphology of the tested phages. As a result of our research, 12 phages specific for *A. baumannii,* 11 of which turned out to be temperate and only one lytic, were isolated. Their lytic spectra ranged from 11 to 75%. The plaques formed by most phages were small and transparent, while one of them formed relatively large plaques with a clearly marked ‘halo’ effect. Based on Transmission Electron Microscopy (TEM), most of our phages have been classified as siphoviruses (only one phage was classified as a podovirus). All phages have icosahedral capsid symmetry, and 11 of them have a long tail. Optimal multiplicity of infections (MOIs) and the adsorption rate were also determined. MOI values varied depending on the phage—from 0.001 to 10. Based on similarities to known bacteriophages, our *A. baumannii*-specific phages have been proposed to belong to the *Beijerinckvirinae* and *Junivirinae* subfamilies. This study provides an additional tool in the fight against this important pathogen and may boost the interest in phage therapy as an alternative and supplement to the current antibiotics.

## 1. Introduction

*Acinetobacter baumannii* is an opportunistic species of Gram-negative bacteria. It poses a serious threat to people staying in hospital wards, especially intensive care units (ICU), as well as people with immunodeficiency [1]. *A. baumannii* is one of the six microorganisms that form the ESKAPE group (*Enterococcus faecium, Staphylococcus aureus*, *Klebsiella pneumoniae*, *A. baumannii*, *Pseudomonas aeruginosa*, *Enterobacter* spp.). These bacteria pose a serious threat both to human health and life due to their growing resistance to commonly used antibiotics. Moreover, the current Centers for Disease Control and Prevention (CDC) report documents a 78% increase in carbapenem-resistant *Acinetobacter* infections [2]. In recent years, the World Health Organization (WHO) has issued reports in which *A. baumannii* has been classified as a critical priority in multi-drug resistant (MDR) bacteria, which may be resistant to third generation cephalosporins and carbapenems, as well as other currently used antibiotics. Genomic analysis of sewage collected from 101 countries around the world (757 sewage samples from 243 cities) in 2016–2019 also confirmed that the problem of transferring antibiotic resistance genes affects the whole world, which poses a threat both to the health and life of animals and humans [3]. The reports noted that the search for new antibiotics against these bacteria should be a priority in today’s research [4,5,6].

Researchers have even distinguished a clear division between antibiotic-resistant strains of *A. baumannii*: ‘XDR’, which are strains resistant to all antibiotics except colistin or tigecycline; ‘MDR’, which are multi-drug resistant or resistant to three or more of the classes of ampicillin-sulbactam, aminoglycoside, antipseudomonal cephalosporin, antipseudomonal carbapenem, fluoroquinolone; and ‘CR’, which are strains resistant to carbapenems, including imipenem and meropenem [7,8].

*A. baumannii* strains can cause various infections, including wound, skin, and soft tissue infections; urinary tract infections; pneumonia; bacteremia; meningitis; and endocarditis [9,10,11]. Among nosocomial infections caused by *A. baumannii*, pneumonia ranks first and mainly affects patients in the ICU. The mortality rate of such infections can be as high as 70% [12,13]. In addition, *A. baumannii* strains are a serious problem in the treatment of burns. Important factors during therapy are the high percentage of MDR *A. baumannii* and the insufficient penetration of antibiotics into damaged tissues, which causes major problems in achieving satisfactory treatment results [14,15,16,17]. Burn infections by *A. baumannii* affects as much as 22% of cases and are most common among military personnel, where the percentage of MDR *A. baumannii* strains reaches up to 53% [18]. Mortality associated with bloodstream infections with this bacterium ranges from 28 to 43% [19]. Interestingly, strains of *A. baumannii* can infect the central nervous system (CNS) and colistin is used to treat these infections [20]. The difficulty in combating infections caused by *A. baumannii*, especially antibiotic-resistant strains, is their ability to survive in the harsh conditions of the external environment, which leads to their increased spread. The extensive use of beta-lactam antibiotics has also contributed to an increase in this phenomenon, necessitating the use of carbapenems in such cases [21], to which resistance has also developed.

In recent years, the interest in phage therapy in the fight against infections caused by *A. baumannii* has increased [22,23]. It has been indicated that *A. baumannii*-specific phages have a high potential to combat bacterial biofilm [24]. Interesting results were obtained by Grygorcewicz et al. (2020) where the AGC01 phage isolated from a fishery pond sample was characterized [25]. Incubation of *A. baumannii* biofilm in the presence of a phage resulted in a reduction of the biomass of the phage sensitive strain AGC01 by about 13%. The results of research on the synergistic effect of phages and antibiotics showed that the best effect was obtained when the phage was combined with ciprofloxacin and meropenem. Other recent studies also point to the combination of phages and ceftazidime being the most effective treatment for bacteremia in mice [26]. Another study using a mouse model of extensively drug-resistant *A. baumannii* bacteremia showed that the using of the φkm18p phage has a positive effect on the survival of mice. In addition, in the group of mice treated with phages, low levels of inflammatory cytokines were observed [27].

In particular, in the era of COVID-19, the overuse of antibiotics caused an increase in observed antimicrobial resistance (AMR) [2]. Interestingly, phages specific for *A. baumannii* can be used in respiratory infections. During the outbreak of the COVID-19 pandemic, a group of researchers treated four patients with critical COVID-19 complicated by pneumonia caused by carbapenem-resistant strains of *A. baumannii*. They were given additional treatment in the form of phage therapy, which brought positive results in the case of two patients who were discharged home [28]. Other studies on the effectiveness of phage therapy in the treatment of respiratory infections with the clinical isolate *A. baumannii* RUH 2037 in a mouse model and in a human lung tissue model showed that mice, after a single intratracheal administration of the lytic phage vB_AbaM_Acibel004, recovered faster than untreated mice and had a lower bacterial burden in the lungs. The bactericidal effect of the purified phage on *A. baumannii* after a single dose was also confirmed in an ex vivo human lung infection model [29]. In 2021, a paper was published presenting the results of phage therapy along with antibiotic therapy in an 88-year-old man who was diagnosed with nosocomial pneumonia caused by carbapenem-resistant *A. baumannii*. The patient was administered a vibrating mesh nebulizer with a personalized specific phage preparation containing the Ab_SZ3 phage for 16 days. Phage therapy was combined with the administration of tigecycline and polymyxin E. This therapy led to improvement in clinical lung function and the removal of the pathogen from the patient’s lungs [30].

It is worth noting that various novel treatment strategies, other than phage therapy, have been recently described that are proposed to combat serious infections. Allemailem et al. (2021) compared the effects of thymoquinone-loaded liposomes and standard antibiotic amoxicillin against drug-sensitive and drug-resistant strains of *A. baumannii* [31]. Interestingly, their results indicate that it is an effective therapeutic formulation in the treatment of infections caused by both types of *A. baumannii* strains. Recent reports also show the efficacy of liposome-based nanovaccine bearing glycosphingolipids from *Sphingomonas paucimobilis* against murine pneumonia associated with *A. baumannii* [32].

In the era of antibiotic resistance, the development of new therapeutic strategies is extremely important. The given examples are quite promising when it comes to phage therapy, especially if the fight against infections caused by MDR strains of *A. baumannii* is considered. Developing research on phages specific for *A. baumannii* strains in the future may lead to the development of an effective therapy against such dangerous infections today.

## 2. Materials and Methods

### 2.1. Bacterial Strains

For the study, 137 strains belonging to *Acinetobacter* spp. (*A. ursingii n* = 1, *A. pitti n* = 2, *A. johnsonii n* = 2, and *A. baumannii n* = 132) and six other Gram-negative strains were used. Bacterial strains were collected from medical environments, thanks to cooperation with hospitals: Regional Specialist Hospital in Wroclaw, University Clinical Hospital in Wroclaw, Military Hospital with Polyclinic in Wroclaw, and Pomeranian Medical University in Szczecin, as well as from the collection of the Bacteriophage Laboratory in the years 2018–2022. Most of the strains showed the resistance to the currently used antibiotics. The species affiliation was determined by matrix-assisted laser desorption/ionization—time of flight (MALDI-TOF/TOF) mass spectrometry (Bruker Daltonics, Billerica, MA, USA). Antibiotic susceptibility was determined by the MicroScan WalkAway analyzer (Beckman Coulter, Brea, CA, USA). All bacterial strains were stored at −70 °C in 25% glycerol. After thawing, the strains were cultured on plates with McConkey agar (peptone 17 g, proteose peptone 3 g, lactose 10 g, bile salts 1.5 g, sodium chloride 5 g, neutral red 0.03 g, crystal violet 0.001 g, agar 13.5 g, water—added to make 1 liter; and pH adjusted to 7.1 +/− 0.2 sodium taurocholate) at 37 °C for 12 h.

### 2.2. Search, Isolation, and Amplification of Bacteriophages

To isolate of bacteriophages specific for *A. baumannii*, 460 samples of water were used (flowing and standing water, water from ponds, rivers, and seas, as well as municipal and hospital wastewater). These samples belonged to the collection of the Hirszfeld Institute of Immunology and Experimental Therapy, Polish Academy of Sciences (HIIET PAS). Liquid samples used for phage screening were stored as samples incubated with peptone water at 37 °C, raw samples, or 20 times concentrated samples (using tangential filtration with the use of a Vivaflow 200 ultrafiltration kit with a Hydrosart membrane and a MWCO 30 kDa operating at a pressure of 3 bar).

The search for bacteriophages was carried out in accordance with the procedure proposed by Ślopek et al. (1984) [33] and is described by Cieślik et al. (2022) [34]. Single colonies of the appropriate bacterial strain were suspended in sugar broth (per 1000 mL of water: meat extract 0.40 g, yeast hydrolysate 1.70 g, NaCl 3.50 g, Bacto Peptone 4.00 g, enzymatic hydrolysate of casein 5.40 g, glucose 10.00 g) and then incubated at 37 °C for 2 h until the optical density at a wavelength of 600 nm (OD_600_) gained in the range of 0.4–0.6 measured spectrophotometrically (BioSpectrometer basic, Eppendorf, Hamburg, Germany). This value corresponds to the logarithmic growth phase of the tested bacteria.

Liquid bacterial cultures were poured onto solid agar plates Tryptone lab-agar ™ (BioMaxima, Lublin, Poland) (per 1000 mL of water: meat extract 3.00 g, Na_2_HPO_4_ 3.00 g, NaCl 5.00 g, tryptone 10.00 g, agar 12.00 g). The collected water samples were applied to the dried plates then incubated at 37 °C overnight and the results were read the next day. Lysis or single plaques on the bacterial lawn surface were considered to be positive results, indicating the probable presence of a phage in the sample. In order to isolate the phage, 200 µL of a 2-h bacterial culture was suspended in 10 mL of peptone water (per 1000 mL of water: meat extract 0.40 g, yeast hydrolysate 1.70 g, NaCl 3.50 g, Bacto Peptone 4.00 g enzymatic hydrolyzate of casein 5.40 g), then 200 µL of a positive water sample was added, mixed, and incubated overnight at 37 °C. After incubation, samples were filtered using syringe filters (with a pore diameter of 0.22 µm, Millipore, Burlington, MA, USA) and Routine Test Dilution (RTD) was performed. For this purpose, serial dilutions of the filtered samples (10^0^–10^−8^) were prepared, and 50 µL of each dilution was applied to pre-prepared plates with a bacterial lawn on the solidified agar surface. The plates were pre-dried at room temperature and then incubated overnight at 37 °C. After incubation, the number of plaques formed at the point of the dilution spot was counted. To determine the titer of the obtained phages, the double-layer agar method was performed [35,36]. For this, 100 µL of a culture of the appropriate bacterial strain and 200 µL of an appropriate phage dilution were added to 2 mL of melted 0.7% agar. The agar with bacteria and phages was mixed and poured onto a solid agar plate. Plates were left for a few minutes at room temperature, incubated overnight at 37 °C, and the titer of bacteriophages was subsequently calculated.

### 2.3. Lytic Spectrum of Examined Phages

In order to determine the lytic spectrum of bacteriophages, 50 µL of the phage (phage titer ~10^7^ PFU/mL (plaque forming unit per milliliter)) was spotted on a plate with the bacterial lawn of the selected bacterial strain previously identified as *Acinetobacter*. The prepared plates were left for a few minutes at room temperature and then incubated at 37 °C overnight. The results were assessed the next day according to the signs: ‘CL’ confluent lysis, ‘SCL’ semiconfluent lysis, ‘OL’ opaque lysis, ‘+++’ confluent plaques >60, ‘++’ 20-60 separate plaques, ‘+’ 10–20 separate plaques, and ‘+/−‘ single plaques. Additionally, species specificity of the obtained phages was performed on strains other than those belonging to *Acinetobacter* spp.: *P. aeruginosa* (*n* = 2), *K. pneumoniae* (*n* = 1), *Escherichia coli* (*n* = 2), and *E. cloacae* (*n* = 1).

### 2.4. Multiplicity of Infection (MOI)

To determine the best parameters that may be obtained during phage amplification, the multiplicity of infection (MOI) was determined for each phage individually. At first, the curves of growth indicating the dependence of the OD_600_ value on the titer of bacteria (phage hosts) were prepared. The previously prepared liquid bacterial culture was diluted 2, 3, 4, 5, 8, and 10-fold. The OD_600_ of the starting sample and of each dilution were measured. Then serial dilutions of each tested sample were made, spotted, and spread on the surface of the agar plate. The plates were incubated overnight at 37 °C. The next day, bacterial colonies were counted, and the titer of the bacterial culture was calculated. On the basis of the obtained data, plots of the dependence of the OD_600_ value on the bacterial titer were prepared.

In the next step, a 2-h bacterial culture was prepared, its optical density (OD_600_) was measured spectrophotometrically, and the bacterial titer was calculated on the basis of the previously prepared graphs. The bacterial liquid culture was diluted to a value of ~10^5^ CFU/mL (colony forming unit per milliliter) and then 200 µL of the diluted bacteria was added to 10 mL of peptone water. The obtained suspension was then incubated for 1 h at 37 °C. Phage dilutions were prepared (10^2^, 10^3^, 10^4^, 10^5^, 10^6^, and 10^7^ PFU/mL) and then 200 µL of each dilution was added to the bacteria (samples with MOI of 0.001, 0.01, 0.1, 1, 10, and 100 were obtained). The samples were incubated overnight at 37 °C and the next day they were filtered, and the phage titer was determined.

### 2.5. Phage Adsorption to Host Bacterial Cells

In order to determine phage adsorption constant and assess the phage adsorption kinetics to bacterial cells, the previously described procedures were used with some modifications [34,37]. In brief, the same volumes of phage lysate and 2-h bacterial suspension (10 mL of each) were mixed at MOI = 0.1 (10^6^ PFU/mL and 10^7^ CFU/mL, respectively). Immediately after mixing and after specific incubation intervals (1, 2, 3, 4, 5, 7.5, 10, 12.5, 15, 17.5, and 20 min) at 37 °C, 1 mL of the sample was taken and filtered. The number of free, non-adsorbed phage particles was determine using the RTD method in triplicate. The number of phages immediately after mixing with the bacterial suspension was considered to be 100% of free phage particles; the other titers were compared to this value. The adsorption rate constants were determined according to Equation (1):

(1) $$k=\frac{2.3}{Bt}log\frac{P0^{\;}}{P}$$

where: k—adsorption rate constant (mL/min); *B*—concentration of bacterial cells; *t*—time interval in which the phage titer falls from *P*0 (original titer) to *P* (final titer) [37].

### 2.6. Transmission Electron Microscopy (TEM)

To check the phage homogeneity in each lysate (whether morphologically homogeneous phages are present) and to assess the morphology and ultrastructure of the obtained phages, the filtered phage lysates in a LB (Luria–Bertani) medium with a titer of 10^7^–10^8^ PFU/mL were prepared for Transmission Electron Microscopy (TEM). The samples were subjected to ultracentrifugation (1 h, 7 °C, 25,000 g), the supernatant was removed, which was followed by 0.1 M ammonium acetate (C₂H₇NO₂) being added and centrifuged again with the same parameters as above. Then, on a 400-mesh copper grid (Athene), a single drop of phage lysate was applied, a contrast in the form of 2% uranyl acetate was added and dried, and the preparation was analyzed using the Zeiss EM900 TEM (at an acceleration voltage 80 kV). Phage photos were obtained using Kodak/Carestream Electron Microscope Film 4489. On the basis of the obtained results, the dimensions and morphology of the examined bacteriophages were determined.

### 2.7. Isolation and Sequencing of Phage DNA

The Genomic Mini AX Phage kit (A&A Biotechnology, Gdansk, Poland) was used to purify and isolate phage DNA from phage lysates with a titer of 10^7^–10^8^ PFU/mL. Phage genomes’ sequencing was outsourced (Biobank Lodz) and Illumina technology was used (Nextera XT DNA Library Preparation, 2 × 250 bp). All phages were subjected to the same general pipeline. Quality assessment was done with the Trimmomatic software [38]. Reads passing the quality check were used by SPAdes software (3.12) to assemble reads into contigs and scaffolds [39,40,41,42,43]. Due to highly fragmented contigs (many repeating sequences in a sample), we first randomly selected enough paired reads to achieve average coverage for contigs with the highest coverage of 6–14 times. Then, such contigs were used as trusted contigs to assemble phage genomes with 20–80 times more paired reads. Assembly artifacts were checked and removed if recognized as bacterial contamination. This allowed one dominant scaffold to be assembled that relates to other phages and this was used as a partial genome for further analysis. BLASTn (megablast) was used to evaluate similarity to Prokaryotic and phage genomes in currently existing databases.

Genome annotations were performed with the Prokka software using Prokaryotic databases [44,45]. We then used a feature of the Genious software (that uses blastn) to improve ORF’s description with the help of genomes of *Acinetobacter* phages that were already accessible (NCBI, ENA, and DDBJ). Then, the most informative description of ORF’s confirmed in this manner was chosen. Such annotated genomes are published as partial genomes through the BankIt software to the NCBI database. GenBank accession numbers for nucleotide sequences are as follows: Acba_1—OQ101250, Acba_3—OQ101248, Acba_4—OQ101249, Acba_6—OQ101251, Aclw_8—OQ101252, Aclw_9—OQ101253, Acba_11—OQ101254, Acba_13—OQ101255, Acba_14—OQ101256, Acba_15—OQ101257, Acba_16—OQ101258, Acba_18—OQ101259.

### 2.8. Phylogenetic Tree

First, we downloaded phage genomes analyzed in a recent *Acinetobacter* phage analysis [46]. Then, we extracted ORF’s that represented major capsid proteins—more precisely ORF’s annotated as major capsid proteins or main capsid proteins. If there were no ORF’s annotated as capsid proteins, we used ones that map to other major capsid proteins present in this analysis. The phylogenetic tree was then calculated by the Genious Prime 2022.1.1 software using the Genious Tree Builder—Tamura-Nei genetic distance model and Neighbour-Joining tree build with method with a 65% similarity matrix as the cost matrix.

## 3. Results

### 3.1. Bacterial Strains Used in the Study

Among the 137 tested strains of *Acinetobacter* spp. used for phage screening, the sources of isolation were: the respiratory tract (49%), urinary tract (23%), infected skin/soft tissues (8%), and blood (4%). Most strains were resistant to currently used antibiotics such as ciprofloxacin, levofloxacin, trimethoprim/sulfamethoxazole, amikacin, gentamicin, netilmicin, tobramycin, tigecycline, colistin, imipenem, or meropenem. *A. baumannii* strains showed the lowest resistance to colistin, however, among all tested strains, six of them were resistant to all the above-mentioned antibiotics. Among the tested strains, apart from *Acinetobacter* spp. strains, other strains of Gram-negative bacteria were used to study the phage species specificity: *P. aeruginosa* (*n* = 2), *K. pneumoniae* (*n* = 1), *E. coli* (*n* = 2), *E. cloacae* (*n* = 1). The list of strains is presented in Table 1, showing the source of strain isolation and their sensitivity to *A. baumannii*-specific phages.

### 3.2. Bacteriophages

#### 3.2.1. General Characteristics

During the tests using liquid samples, a total of 18 bacteriophages specific for *A. baumannii* strains were obtained; however, genetic studies revealed that out of all 18 bacteriophages, 12 are individual phages that could have been used in further research (Table 2). Six of them were previously deposited in the collection of the Bacteriophage Laboratory (2007–2015).

#### 3.2.2. Morphology and Dimensions of Phages and Their Plaques

**Figure 1:**
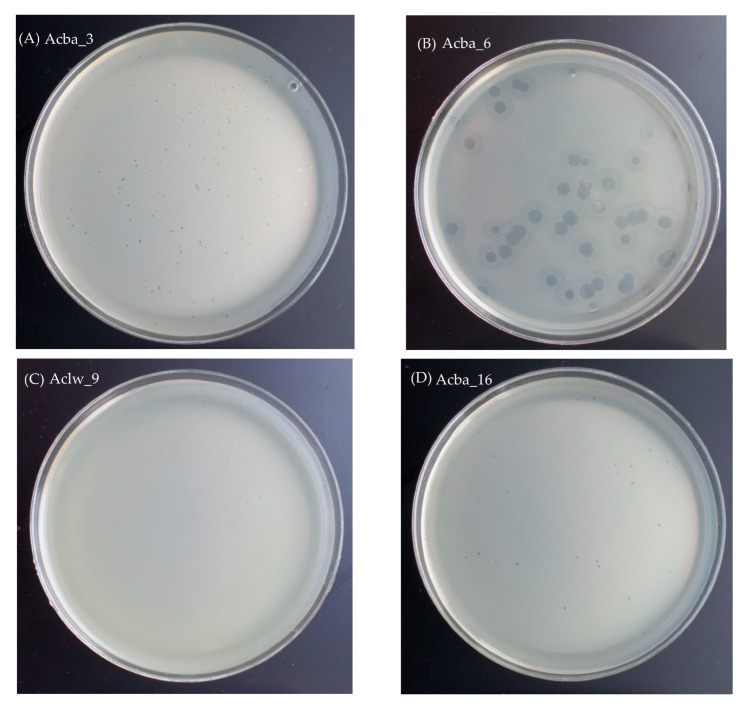
Pictures showing the morphology of plaques of selected phages. Panel (**A**): Acba_3, Panel (**B**): Acba_6, Panel (**C**): Aclw_9, Panel (**D**): Acba_16. Small, transparent plaques (Acba_3, Aclw_9, Acba_16) and larger plaques with a ‘halo’ effect (Acba_6) are visible on the plates. Photos of phage plaques were taken with the Samsung ST150F camera.

**Figure 2:**
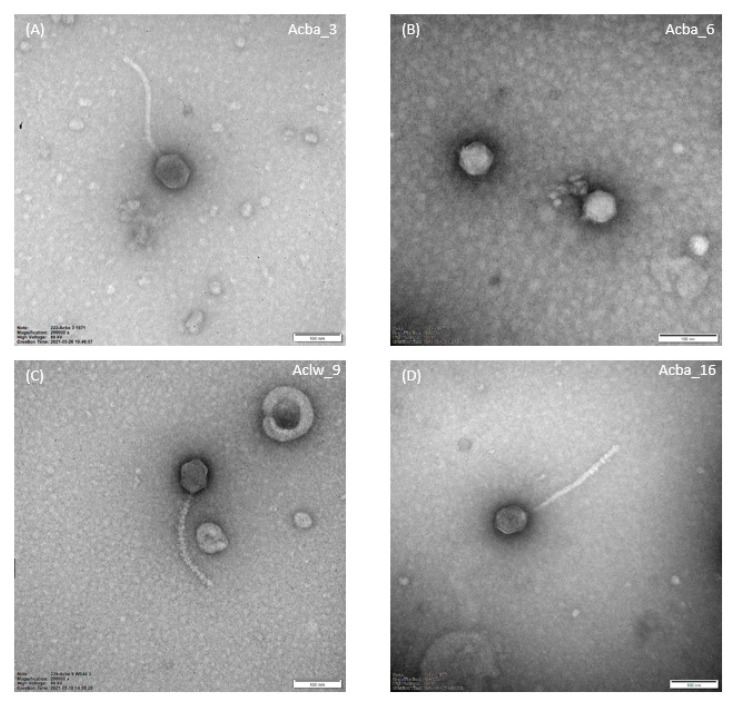
Electron micrographs from the TEM (Kodak/Carestream Electron Microscope) showing the morphology of selected bacteriophages. Panel (**A**): Acba_3, Panel (**B**): Acba_6, Panel (**C**): Aclw_9, Panel (**D**): Acba_16. The Acba_3, Aclw_9, and Acba_16 phages were photographed at a magnification of 200,000×, whereas the magnification for the Acba_6 phage was 250,000×. The reference bar shown on each panel corresponds to 100 nm.

Nine out of 12 phages formed small transparent plaques 0.5–1 mm in diameter, Aclw_8 and Aclw_9 formed small transparent plaques ~0.5 mm in diameter, and Acba_6 phages formed transparent plaques 3–4 mm in diameter with a clearly visible ‘halo’ effect. Images of plaques of selected phages are shown in the Figure 1, while pictures from the TEM are shown in Figure 2. The photos presenting plaque morphology and electron micrographs of the remaining phages are included in the Supplementary Materials (Figures S1 and S2). Electronograms obtained with the use of TEM allowed to visualize the virion morphology. All phages have icosahedral capsid symmetry, and all phages except Acba_6 have a long tail. For phages Aclw_8, Aclw_9, Acba_11, Acba_13, Acba_16, and Acba_18, transverse rings along the tail are visible (Figure 2, Supplementary Figure S2). Phage virion measurements were made on the basis of electron micrographs; total dimension, capsid length, capsid width, capsid diagonal, tail length, and tail width were determined (Table 3). The total dimension for 11 out of 12 phages was ~300 nm, while for the Acba_6 phage this dimension was 56.9 nm (Table 3).

### 3.3. Investigating the Best MOI to Optimize Phage Amplification

Different ratios of phage titer to bacterial titer (MOI) were studied to optimize bacteriophage amplification. Our research revealed that the tested *Acinetobacter* phages amplified optimally at different MOIs. During the study of the best MOI, the bacterial titer in each case was the same and was 10^5^ CFU/mL. The obtained results that lead to individual phages obtaining the highest titer are shown in the Table 4.

### 3.4. Phage Adsorption to Bacterial Cells

**Figure 3:**
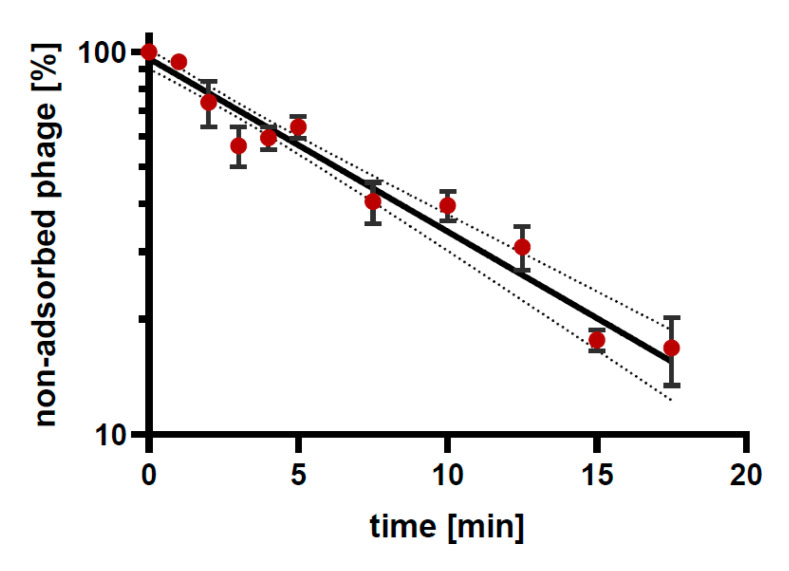
Kinetic of Acba_6 adsorption on *A. baumannii* 3940 at MOI = 0.1. Points represent an average; error bars represent the standard deviation of the mean phage titers for three samples (± SD). Lines correspond to the model (linear regression) used to calculate the adsorption constant; dotted line is the 95% confidence interval (CI).

As a result of studies on the infection of bacterial hosts with individual phages, the level of adsorption of phage particles to bacterial cells at MOI = 0.1 at 37 °C was between 38% (for Aclw_9 phage and *A. baumannii* 703 strain) and 82% (for Acba_6 phage and their host *A. baumannii* 3940). The time required to adsorb the largest number of phage particles also differed in individual cases and ranged from 3 min for Acba_15 phage and 4 min for both Aclw_9 and Acba_16 phages, to 17.5 min for Acba_3 and Acba_4 bacteriophages. A graph showing an example of the kinetics of Acba_6 phage adsorption to their bacterial hosts (*A. baumannii* 3940) is shown in Figure 3. The calculated adsorption rate constants for each phage are presented in Table 5.

### 3.5. Genome Sequencing

We present the genomes of 12 new *Acinetobacter* phages (Table 6). All phages were sequenced with Illumina technology and assembled into contigs from reads passing through a quality check (see Materials and Methods for details). Phages Acba_3, Acba_4, Acba_6, Aclw_9, Acba_11, Acba_13, Acba_14, Acba_15, and Acba_18 assembled into gapless scaffolds, phages Acba_1, Aclw_8, and Acba_16 contain assembly gaps. Researched *Acinetobacter* phages were annotated by Prokka, BLAST, and Geneious2022 software (see Section 2 for details). We determined between 53 (Aclw_9) to 96 (Acba_11) ORF’s per phage. Proteins not described as ’hypothetical proteins’ account for between 41% (Aclw_9) to 65% (Acba_6) of ORFs (Table 6). No tRNA’s coding sequences were detected (as determined by ARAGON as part of the Prokka software).

All phages except Acba_6 (which we will describe separately) show varying degrees of similarity to each other with very high similarity (over 95% pairwise identity) in similar fragments. Despite this, presented genomes are extremely poorly represented on the level of the nucleotide sequence by *Acinetobacter* phages in currently available databases as determined by megablast. Most presented phages show a similarity to other phages in less than 50% of a genome. For most presented phages, the most similar phage is either *Acinetobacter* phage AM106 (acc. no.: MH115576.1) or YMC11/11/R3177 (acc. no. NC_041866). Most similar to the already published phages, the Aclw_8 phage shows over 50% similarity in the genome’s length to five other known phages with a maximum of 65% nucleotide identity to *Acinetobacter* phage Ab11510-phi (acc. no.: MT361972.1). With higher similarity to the presented phages genomes, *A. baumannii* bacteria’s prophage genomes had around 80% nucleotide identity for the best hit. This is still relatively far from the similarity cut-off required for different phage species (95% of nucleotide identity) showing how poorly *Acinetobacter* phages are currently represented in databases. Still, despite the differences, we found no unique proteins or genes. Interestingly, *Acinetobacter* phage Acba_6 shows little similarity to other presented phages and *Acinetobacter* phage vB_ApiP_P1 (acc. no.: NC_042006.1) is the closest relative (95% query cover), but no significant similarity to prophages in *A. baumannii* phage genomes was found by megablast.

Based on previous work attempting to classify *Acinetobacter* phages and the International Committee on the Taxonomy of Viruses (ICTV) recommendations [46,47,48], we propose a taxonomical classification of the presented phages (Table 7). For a detailed relationship supporting the proposed taxonomical classification, we present the phylogenetic tree of the major capsid protein for *Acinetobacter* phages and the presented phages (Supplementary Data S1). In the current work, we use the classification and representative genomes proposed recently and follow this nomenclature in subsequent parts of our work [46].

**Figure 4:**
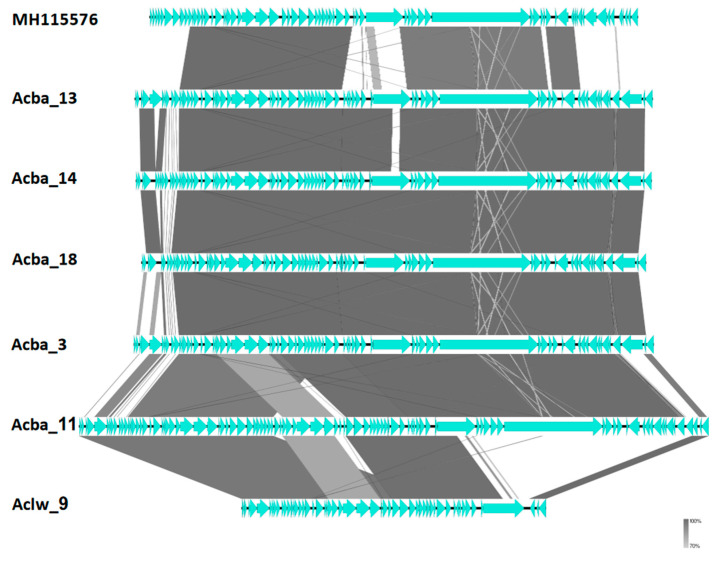
Comparison of genomes between researched phages and the reference phage for *Shemyakinvirus* genus. Genomes were compared using blastn and represented by the Easyfig software. The genome of the representative phage is on the top. Horizontal black lines (genome) with blue arrows (ORFs) represent phage genomes. Gray, thick, vertical lines represent similar fragments of genomes between phages. Thin gray or white lines represent similarity between short, internal repeats found in compared genomes.

**Figure 5:**
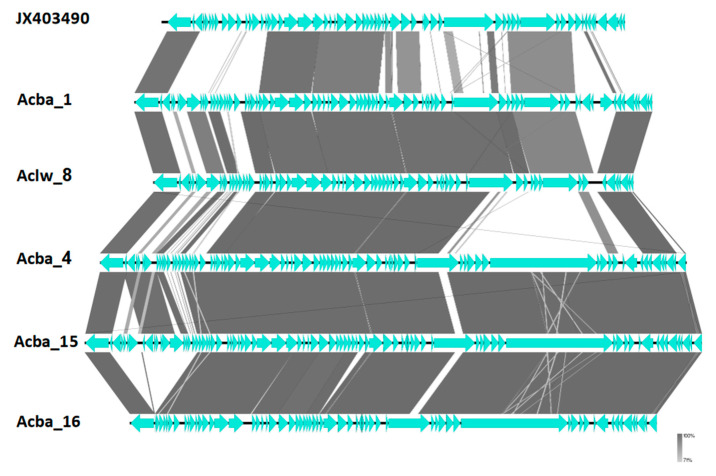
Comparison of genomes between researched phages and the reference phage for *Vieuvirus* genus. Genomes were compared using blastn and represented by the Easyfig software. The genome of the representative phage is on the top. Horizontal black lines (genome) with blue arrows (ORFs) represent phage genomes. Gray, thick, vertical lines represent similar fragments of genomes between phages. Thin gray or white lines represent similarity between short, internal repeats found in compared genomes.

**Figure 6:**
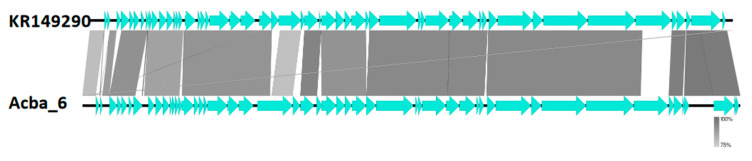
Comparison of genomes between researched phage Acba_6 and a reference phage from *Friunavirus* genus. Genomes were compared using blastn and represented by the Easyfig software. Horizontal black lines (genome) with blue arrows (ORFs) represent phage genomes. Gray, thick, vertical lines represent similar fragments of genomes between phages. Thin gray or white lines represent similarity between short, internal repeats found in compared genomes.

Despite low similarity on a nucleotide level, we show that genome organization and ORF composition is relatively similar across representatives of proposed subfamilies and genera (Figure 4, Figure 5 and Figure 6). The Acba_6 phage fits firmly in the well represented *Friunavirus* genus in the *Beijerinckvirinae* subfamily. We propose that phages Acba_3, Aclw_9, Acba_18, Acba_14, Acba_11, and Acba_13 to be classified as *Shemyakinvirus* in the *Junivirinae* subfamily [46]. The Acba_13 phage is relatively distant from other phages and can create a different genus in the future, but importantly, a more detailed analysis and classification would be required to determine that. We propose that phages Acba_1, Acba_16, Acba_4, Acba_15, and Aclw_8 to be classified as *Vieuvirus* in the *Junivirinae* subfamily. For all phages, ends were fitted to representative phages of their respective, proposed genera.

For *Acinetobacter* phage Acba_6 we found no integrase protein, CI repressor protein, or telomere regulation protein, which suggests a lytic cycle for this phage. A CI repressor protein was found in the Aclw_8 and Aclw_9 phages and in the rest of the presented phages we found an integrase protein. In all phages except Acba_6, we also found a telomere regulation protein (similar to protein UFJ83265.1 in *Acinetobacter* phage Ab1656-2, acc. no: MZ675741.1).

## 4. Discussion

Due to the serious threat to the health and life of people around the world from the *A. baumannii* species, we have undertaken research aimed at isolating and characterizing phages specific to these dangerous drug-resistant strains. In this paper, we present the results of studies on biological properties such as: the morphology of plaques, phage virion morphology and ultrastructure, their lytic spectrum on strains of *A. baumannii* and lytic activity on other strains of Gram-negative bacteria, the kinetics of phage adsorption to host cells, as well as optimal amplification parameters (herein MOI). Most importantly, we conducted genetic studies, which are now the basis of phage research specifically intended for therapy [49,50]. This enabled the assessment of the phages’ therapeutic potential and, on this basis, it was possible to propose the classification our *Acinetobacter* phages to the appropriate genus and family.

The bacterial strains used in the research largely came from patients` respiratory and urinary tracts, with most strains showing resistance to the currently most commonly used antibiotics. *A. baumannii* strains belong to the *Acinetobacter calcoaceticus-baumannii* (Acb) complex. This complex consists of five pathogenic and one non-pathogenic species: *A. baumannii*, *A. pittii*, *A. nosocomialis*, *A. seifertii*, *A. dijkshoorniae,* and *A. calcoaceticus*. These species show phenotypic and genotypic similarity [51]. As published elsewhere, the genome of *A. baumannii* is characterized by plasticity and dynamism (despite the limited variability of the core genes), which is manifested in the significant variability of the accessory gene, which results from the acquisition of a wide variety of mobile genetic elements by the bacterial strains [52]. Analysis of bacterial isolates from the international clonal lineage I showed that the targets of phage adsorption are often hotspots for recombination combined with outer membrane proteins and a capsular polysaccharide, lipooligosaccharide (LOS) [53,54].

In connection with the above, phages may be an effective solution to the aforementioned problems and threats from *A. baumannii*. Our aim was to isolate and characterize the phages with therapeutic potential. From environmental and clinical material, we initially obtained 18 probable phages; however, the genome analysis revealed that 12 of them were different/individual phages, and only one of them showed the characteristics of a lytic phage with therapeutic potential. It is likely the observed difficulties both with the isolation of these phages and their amplification to titers higher than 10^8^ PFU/mL may be due to the fact that most of them are temperate. Interestingly, some authors proposed the possibility of prophage induction therapy, after appropriate modifications of temperate phages [55]. Therefore, we decided to describe in detail all the isolated phages despite their temperate nature.

Our findings show that all the isolated phages were species-specific—they lysed only *Acinetobacter* strains without affecting other selected Gram-negative bacteria (Table 1, Table 2). Their lytic spectrum (Table 2) on tested *A. baumannii* strains can be considered narrow and moderate: from 11% (Acba_1 and Acba_18) through to 30% (Acba_4) and to a maximum of 75% (Acba_15). For half of the 12 phages, alternative hosts strains have been found in addition to the main host on which the phages were isolated (Table 2). Jin et al. (2012), who also studied the range of activity of a phage (ZZ1) specific for *A. baumannii* strains, observed that apart from the host strain (AB09V), two additional strains (AB0901 and AB0902) from 22 tested clinical isolates were found to be particularly sensitive to phage titer changes, both of which showed lysis of the bacterial lawn when using phage titers of 10^7^–10^8^ PFU/mL or higher [56]. In another study, out of 14 phages specific for *A. baumannii*, the phage with the widest lytic spectrum was PD-6A3, for which the host range was ~32%. For comparison, a phage cocktail composed of all 14 bacteriophages lysed 54% of all tested bacterial strains [57].

Plaque morphology for most phages was similar—the plaques formed by the phages were small (0.5–1 mm in diameter) and transparent. Phage Aclw_8 and Aclw_9 had a slightly smaller plaque diameter (approximately 0.5 mm), while phage Acba_6 was characterized by relatively large plaques with a clearly marked ‘halo’ effect (Figure 1). The morphology of the phage virions is shown in Figure 2. While most of our phages have been classified as siphoviruses (only one phage was classified as a podovirus), *Acinetobacter*-specific phages are also often described as myoviruses [50,58,59]. The total dimension for 11 out of 12 phages was approximately 300 nm, while for the Acba_6 phage this dimension was 56.9 nm. Each of the phages had a head with icosahedral symmetry. Additionally, 11 out of 12 phages had a long tail, except for the Acba_6 phage, where the virion had no tail. Interestingly, Acba_6 shows similarities to the *A. baumannii* φkm18p phage, which also has no tail, and its hexagonal head is about 59 nm in diameter [7]. In another paper describing phages active against *A. baumannii,* there are micrographs of eight phages with symmetry icosahedral heads of approximately 60 nm in diameter and short noncontractile tails of 10 nm in length [60]. Other examples of *A. baumannii*-specific phage morphology are the ISTD and NOVI phages. Both phages had a long, contractile, and thick tail with protruding tail tubes. The NOVI phage head was isometric, while the ISTD phage head was more elongated [61].

MOI studies (Table 4) have shown that the optimal parameter needed for phage amplification is an individual issue for each *Acinetobacter* phage. For six out of 12 phages, the optimal MOI oscillates between 0.001 (Acba_1, Acba_3, and Acba_6) and 0.1 (Acba_4, Aclw_8, and Acba_11). For five phages, the optimal MOI was 1 (Aclw_9, Acba_13, Acba_14, Acba_16, and Acba_18), and for one phage the MOI was 10 (Acba_15). For some phages, lower values of MOI (MOI = 0.1) are more effective [60,62], which is also the case for phages against other species, as described for *Enterobacter*-specific bacteriophages [34]. For other phages, the highest phage titer values will be obtained with a higher MOI, for example, an MOI of 20 [8].

The fact that the studied phages are temperate has been confirmed by the presence in the analyzed genomes of both integrase and CI repressor genes. The presence of the integrase gene in the phage genome may indicate that these phages carry out the lysogenic [63]. Because integrases may be, e.g., associated with the recombination and to the genomic rearrangements [24], which result in integration and excision of phage genomes from the host genome, the maintenance of the number of plasmid copies, or elimination of chromosomal dimers [64]. The CI repressor protein plays a key role in the regulation of the lytic/lysogenic cycle of phages. This protein inhibits the transcription of the pL lytic promoter by binding to multiple operator sites in the DNA [65]. The prophage genome integrates with the bacterial chromosome, which leads to the creation of a stable form of the prophage present in the bacterial genome in the next generations [66]. Interestingly, the presence of both integrase and the telomere regulation protein may be used in the mechanism when phages enter the lysogenic cycle as linear plasmids inside the bacteria with covalently closed hairpin ends [67]. As was described elsewhere, the genomes of these prophages consist of two arms, which are separated by the telomere resolution site (essential for phage genome conversion into the linear plasmid prophage).

Based on the published papers from 2017 to 2021, in the International Nucleotide Sequence Database Collaboration, the number of sequenced *Acinetobacter*-specific phage genomes increased from 37 to 139 [46]. These phages, due to their genetic diversity, can be assigned to eight clusters (subfamilies). The authors proposed a new solution in the classification of *Acinetobacter* phages: reorganization of the genus *Obolenskvirus* and the creation of five new subfamilies [46]. The Bacterial Viruses Subcommittee of the ICTV currently recommend to use genome-based metrics to determine the taxonomic affiliation of phages. Interestingly, genetic analyzes of lytic phage genomes have shown that their evolution is predominantly vertical, and some of the clades of phages represent ancient lineages [47].

In a phylogenetic analysis, only one protein was used, as using all proteins would cause classification problems because *Acinetobacter* phages are difficult to classify. The pan-proteomic analyses of clusters used for this (subfamilies from, e.g., work by Oliveira et al. (2022) [46]) are based on different rules than the ICTV guidelines, which additionally do not take many phages into account. This creates problems when a larger number of proteins is analyzed.

Nowadays, when there is a trend suggesting the possibility of using temperate phages in the therapy of bacterial infections, we present data on the biological properties of *Acinetobacter* phages, which may be used for therapeutic purposes in the future after proving their safety and effectiveness. Alternatively, or perhaps additionally, it creates the possibility of using the potential of enzymes encoded in the genome of phages.

## 5. Conclusions

In the above study, we have examined the biological properties of 12 phages specific for *A. baumannii*, including the morphology of plaques, phage virion morphology and ultrastructure, their lytic spectrum, the kinetics of phage adsorption to host bacterial cells and kinetics rate constants, as well as optimal MOI. Genetic studies also allowed us to check the presence of genes characteristic of the phage lytic cycle. Moreover, we conducted genetic studies, which allowed us to classify the phages to the appropriate genus and family. The above research will allow us to supplement the knowledge about phages specific for *A. baumannii* strains, which currently pose a serious threat to human life and health around the world. In addition, attention should be paid to phage therapy, which may become a serious alternative to the currently widely used antibiotic therapy. In order to assess the therapeutic potential, additional stability studies of the described phages are underway, which we also intend to publish.

Nevertheless, the data presented in this study broadens the spectrum of phages applicable in the fight against this important pathogen and contribute to the further advancement of phage therapy.

## Figures and Tables

**Table 1:** Characteristics of Gram-negative bacterial strains other than *Acinetobacter* spp.

Bacterial Species	Source of Isolation	Sensitivity to Tested *A. baumannii* Phages
*P. aeruginosa* 6111	swab from the fistula	no
*P. aeruginosa* 1790/1	sputum	no
*K. pneumoniae* 8385	urine	no
*E. coli* 9608	vagina	no
*E. coli* 8069	urine	no
*E. cloacae* 2084	urine	no

**Table 2:** Characterization of phages specific for *A. baumannii*. The lytic spectrum was tested with 53 strains of *A. baumannii*.

No.	Phage Symbol	ICTV Symbol	Source and Year of Isolation	Lytic Spectrum	Host Strain
1.	Acba_1	*Acinetobacter* phage vB_AbaS-1	city ditch water (Sroda Slaska, Lower Silesia, Poland, 2007)	11%	*A. baumannii* K26905 (main) * A. baumannii: 703, 1326, 1971 (alternative) **
2.	Acba_3	*Acinetobacter* phage vB_AbaS-3	water sample (Kluczowa, Lower Silesia, Poland, 2018)	47%	*A. baumannii* 1971
3.	Acba_4	*Acinetobacter* phage vB_AbaS-4	water sample (Drobnice, Lodz Province, Poland, 2018)	30%	*A. baumannii* 1971
4.	Acba_6	*Acinetobacter* phage vB_AbaP-7	hospital sewage (delivery and operating block) Regional Specialist Hospital in Wroclaw (Wroclaw, Lower Silesia, Poland, 2013)	19%	*A. baumannii* 3940
5.	Aclw_8	*Acinetobacter* phage vB_AlwS-8	sewage treated from a sewage treatment plant (Grodzisk Mazowiecki, Masovian, Poland, 2014)	11%	*A. lwoffi* WII44.3 (main) *A. baumannii*: 703, 1326, 1971 (alternative)
6.	Aclw_9	*Acinetobacter* phage vB_AlwS-9	water taken from the Water Treatment Plant (Grodzisk Mazowiecki, Masovian, Poland, 2015)	72%	*A. lwoffi* WII44.3 (main) *A. baumannii*: 703, 1326, 1971 (alternative)
7.	Acba_11	*Acinetobacter* phage vB_AbaS-11	water sample (Walichnowy, Lodz Province, Poland, 2018)	62%	*A. baumannii* 1326
8.	Acba_13	*Acinetobacter* phage vB_AbaS-13	river water (Olawa, Lower Silesia, Poland, 2018)	17%	*A. baumannii* 1326
9.	Acba_14	*Acinetobacter* phage vB_AbaS-14	water sample (Walichnowy, Lodz Province, Poland, 2018)	21%	*A. baumannii* 1971
10.	Acba_15	*Acinetobacter* phage vB_AbaS-15	water sample (Lyskornia, Lodz Province, Poland, 2018)	75%	*A. baumannii* 703 (main) *A. baumannii*: 1326, 1971 (alternative)
11.	Acba_16	*Acinetobacter* phage vB_AbaS-16	water sample (Lyskornia, Lodz Province, Poland, 2018)	13%	*A. baumannii* 703 (main) *A. baumannii*: 1326, 1971 (alternative)
12.	Acba_18	*Acinetobacter* phage vB_AbaS-18	water from the drainage ditch (Olawa, Lower Silesia, Poland, 2018)	11%	*A. baumannii* 703 (main) *A. baumannii*: 1326, 1971 (alternative)

* the bacterial strain on which the phage was isolated. ** the bacterial strain on which the phage may be amplified.

**Table 3:** The dimensions of the bacteriophages were determined based on the measurements of ten virions for each of 12 phages.

Phage Symbol	Total Dimension [nm]	Capsid Length [nm]	Capsid Width [nm]	Capsid Diagonal [nm]	Tail Length [nm]	Tail Width [nm]
Acba_1	316.9	64.8	64.2	67.6	252.1	10.7
Acba_3	331.5	82.8	65.4	77.2	248.7	10.1
Acba_4	292	68.5	54.6	65.7	223.5	9.8
Acba_6	56.9	56.9	58.6	n/a	n/a	n/a
Aclw_8	308.8	71.5	56.3	67.2	237.3	9.7
Aclw_9	307.3	76.1	59.7	67.1	231.2	10.3
Acba_11	315.9	67.9	56.1	65.9	248	9.4
Acba_13	329	76.2	62.8	75.2	252.8	10.3
Acba_14	316	78.7	65.8	74.5	237.3	9.3
Acba_15	322.5	78.3	61.6	73.3	244.2	10.3
Acba_16	319.4	73.6	58.3	68.8	245.8	9.6
Acba_18	297.1	64.6	52.8	61.1	232.5	9.4

**Table 4:** MOI values resulting in the highest titer of the tested phages.

Phage Symbol	Phage Titer [PFU/mL]	MOI	Phage Titer after Incubation [PFU/mL]
Acba_1	10^2^	0.001	7.89 × 10^6^
Acba_3	10^2^	0.001	9.76 × 10^6^
Acba_4	10^4^	0.1	4.73 × 10^7^
Acba_6	10^2^	0.001	2.31 × 10^8^
Aclw_8	10^4^	0.1	1.86 × 10^7^
Aclw_9	10^5^	1	5.16 × 10^7^
Acba_11	10^4^	0.1	4.74 × 10^7^
Acba_13	10^5^	1	9.97 × 10^6^
Acba_14	10^5^	1	4.73 × 10^7^
Acba_15	10^6^	10	6.16 × 10^7^
Acba_16	10^5^	1	4.61 × 10^7^
Acba_18	10^5^	1	7.71 × 10^7^

**Table 5:** Adsorption rate constants [mL/min] for 12 *Acinetobacter*-specific bacteriophages.

Adsorption Rate Constant [mL/min]	
Acba_1	1.6 × 10^−9^
Acba_3	1.2 × 10^−9^
Acba_4	1.33 × 10^−8^
Acba_6	5.76 × 10^−9^
Aclw_8	1.04 × 10^−9^
Aclw_9	2.61 × 10^−9^
Acba_11	4.6 × 10^−9^
Acba_13	1.11 × 10^−9^
Acba_14	4.8 × 10^−9^
Acba_15	2.6 × 10^−9^
Acba_16	1.66 × 10^−8^
Acba_18	5.6 × 10^−9^

**Table 6:** Basic features of sequenced phages.

Phage Symbol	Genome Length [bp]	No. of ORFs	Strand [%]		Described Protein [%]	Start Codons [%]		
			Positive	Negative		ATG	TTG	GTG
Acba_1	50,696	77	79.2	20.8	51.9	90.9	2.60	5.19
Acba_3	55,181	72	80.6	19.4	52.8	97.2	1.39	1.39
Acba_4	57,429	77	81.8	18.2	51.9	93.5	2.60	3.90
Acba_6	42,776	52	100.0	0.0	65.4	98.1	1.92	0.00
Aclw_8	47,018	70	84.3	15.7	45.7	94.3	1.43	4.29
Aclw_9	32,415	53	92.5	7.5	41.5	94.3	1.89	3.77
Acba_11	67,052	96	84.4	15.6	47.9	95.8	1.04	3.13
Acba_13	55,385	73	80.8	19.2	49.3	98.6	1.37	0.00
Acba_14	54,941	74	81.1	18.9	44.6	94.6	2.70	2.70
Acba_15	60,424	81	80.2	19.8	53.1	95.1	2.47	2.47
Acba_16	51,595	64	81.3	18.8	50.0	95.3	1.56	3.13
Acba_18	53,751	70	80.0	20.0	48.6	98.6	1.43	0.00

**Table 7:** Proposed taxonomy of the presented genomes on the basis of nucleotide sequence similarity and phylogenetic analysis of the major capsid protein in *Acinetobacter* phages. Presented classification, cluster designation, and representative phages are described in the cited work [46].

Presented Phage	Most Similar and Representative Phages		Subfamily (Proposed)	Taxonomy (Proposed)	Cluster
	Acc. no.	NCBI Description			
Acba_1 Acba_16 Acba_4 Acba_15 Aclw_8	NC_041866 NC_019541	*Acinetobacter* phage YMC11/11/R3177 *Acinetobacter* phage YMC/09/02/B1251_ABA_BP	*Junivirinae*	*Vieuvirus*	F3
Acba_3 Aclw_9 Acba_18 Acba_14 Acba_11 Acba_13	MH115576	*Acinetobacter* phage AM106	*Junivirinae*	*Shemyakinvirus*	F1
Acba_6	MN604239 NC_042007 KR149290	*Acinetobacter* phage vB_AbaP_APK87 *Acinetobacter* phage vB_ApiP_P2 *Acinetobacter* phage Fri1	*Beijernickvirinae*	*Friunavirus*	E2

## Data Availability

The presented data are available on request from the corresponding author.

## Supplementary Materials

The following supporting information can be downloaded at: https://www.mdpi.com/article/10.3390/v15010231/s1, Figure S1: Pictures presenting the morphology and size of phage plaques; Figure S2: Electron micrographs from the Transmission Electron Microscope showing the morphology of bacteriophages; Data S1: *Acinetobacter* phages major capsid protein tree.
