# Supplementary material for: Biological Properties of 12 Newly Isolated Acinetobacter baumannii-Specific Bacteriophages
Source: Viruses. 2023 Jan 13;15(1):231. doi: 10.3390/v15010231 (PMC9866556; doi:10.3390/v15010231)
Supplement: Supplementary file 1 [file viruses-15-00231-s001.zip › Figure S1.docx]

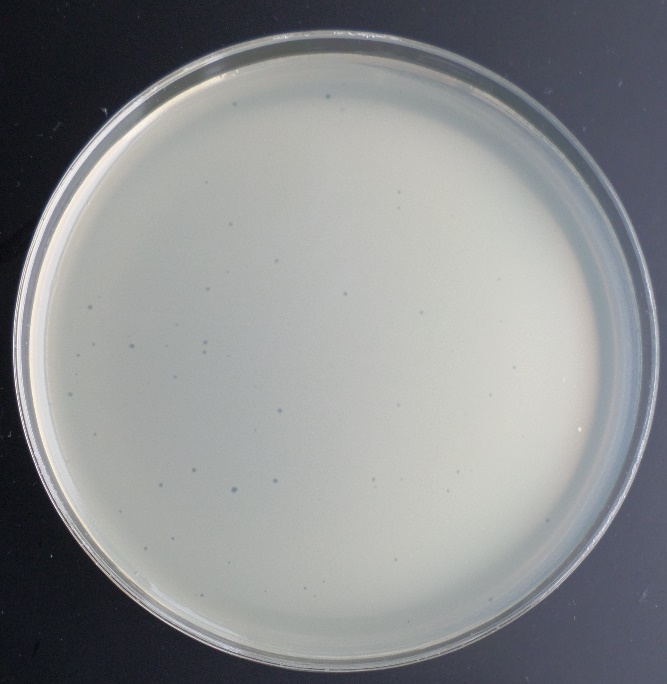

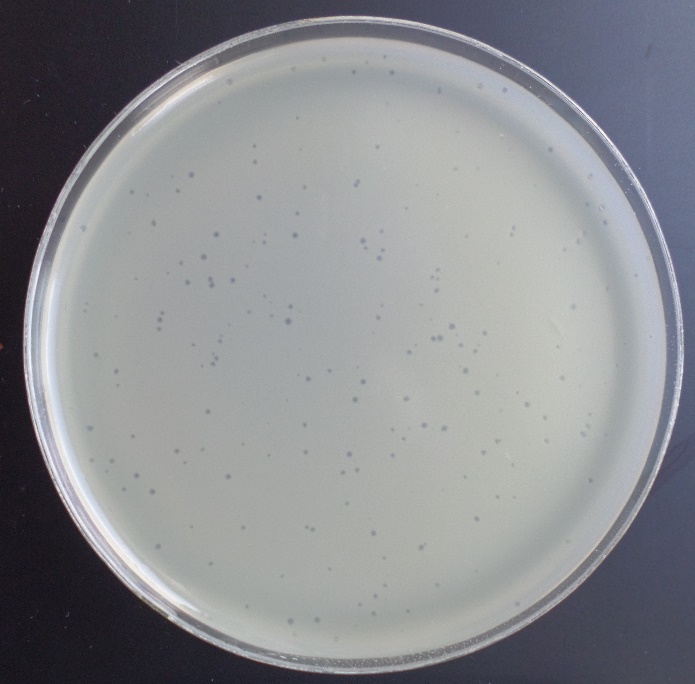


Acba_4

(B)

Acba_1

(A)


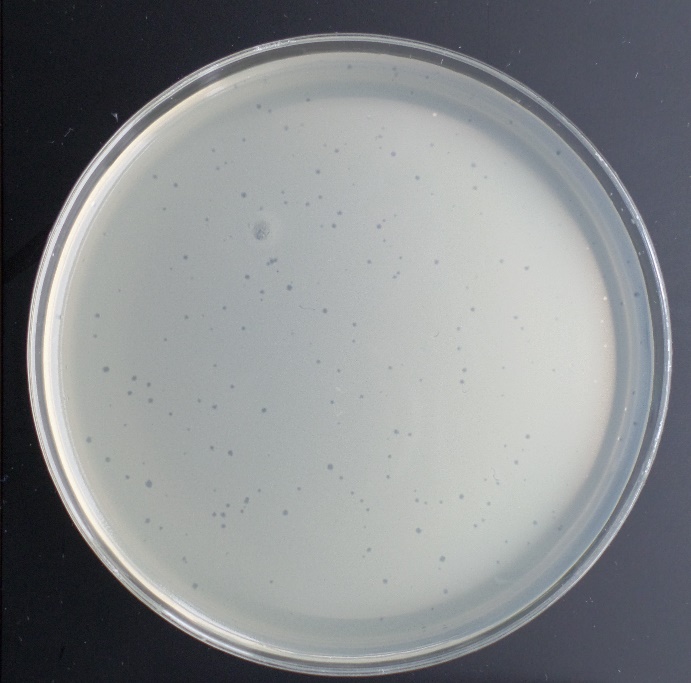

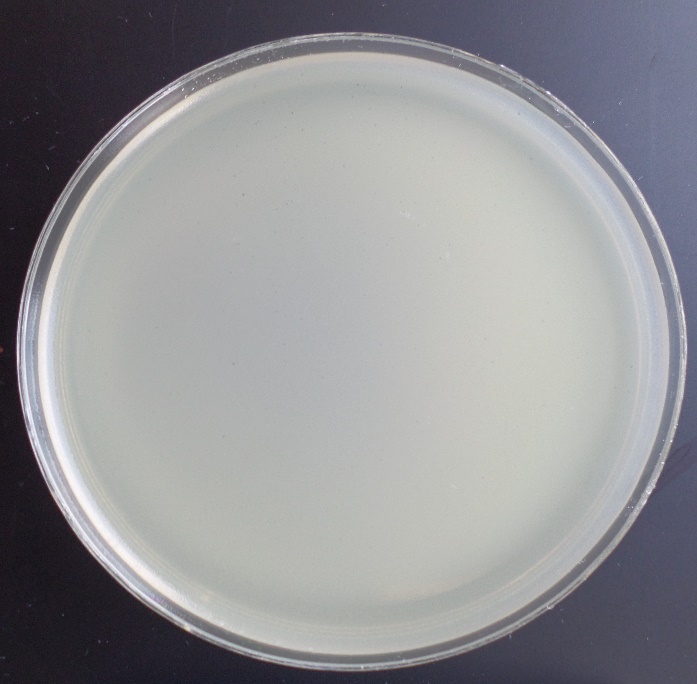


(D)

(C)

Acba_11

Acba_8


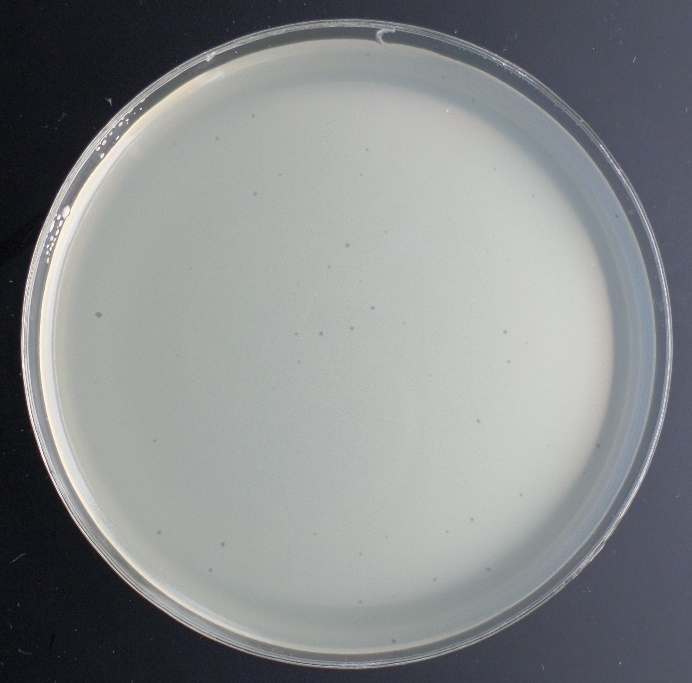

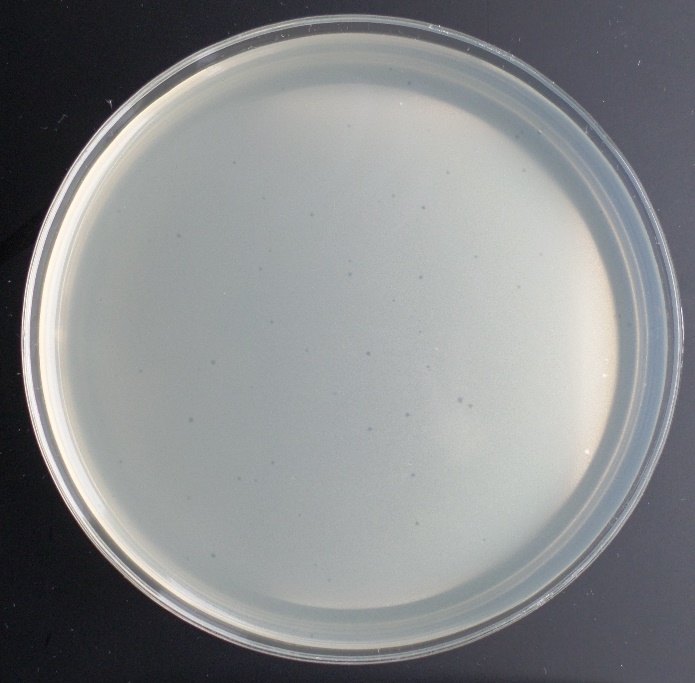


(F)

(E)

Acba_13

Acba_14


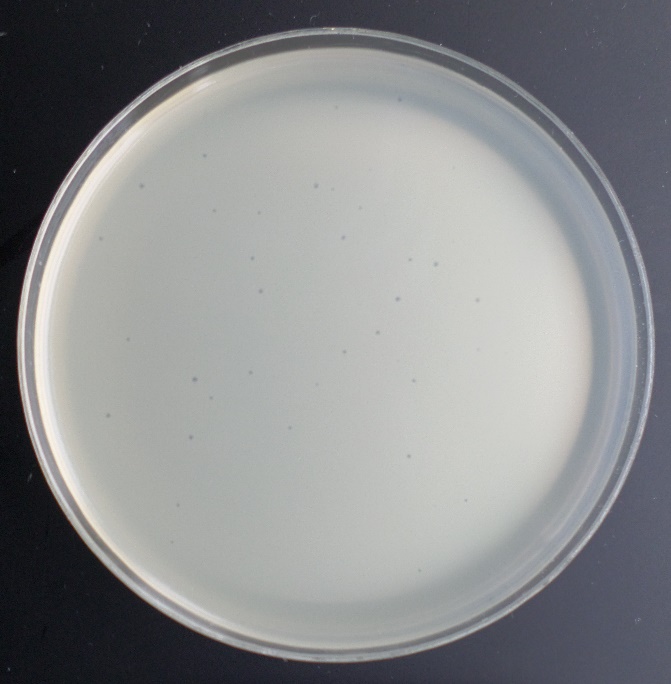

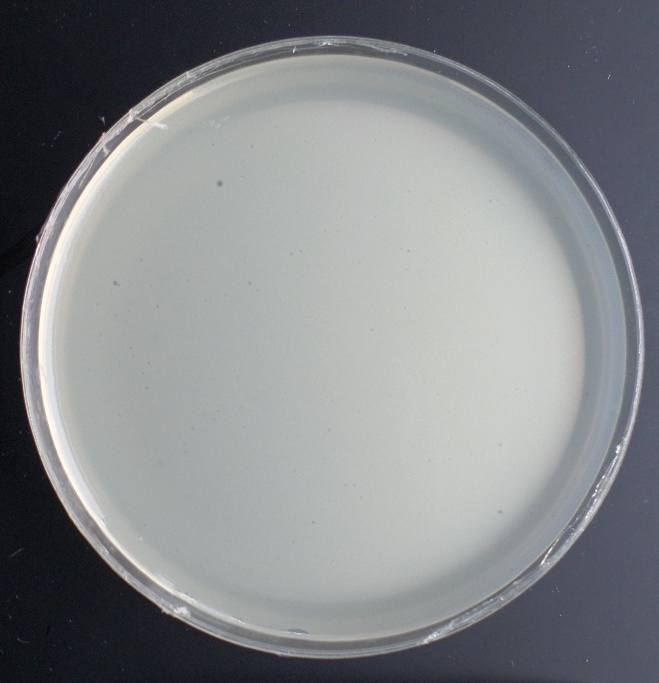


(H)

Acba_15

(G)

Acba_18

Figure S1. Pictures showing the morphology of plaques of selected phages. Panel (**A**): Acba_1, Panel (**B**): Acba_4, Panel (**C**): Acba_8, Panel (**D**): Acba_11, Panel (**E**): Acba_13, Panel (**F**): Acba_14, Panel (**G**): Acba_15, Panel (**H**): Acba_18. Small (~0.5-1 mm diameter), transparent plaques are visible on the plates. Photos of phage plaques were taken with the Samsung ST150F camera.
