# Supplementary material for: Biological Properties of 12 Newly Isolated Acinetobacter baumannii-Specific Bacteriophages
Source: Viruses. 2023 Jan 13;15(1):231. doi: 10.3390/v15010231 (PMC9866556; doi:10.3390/v15010231)
Supplement: Supplementary file 1 [file viruses-15-00231-s001.zip › Figure S2.docx]

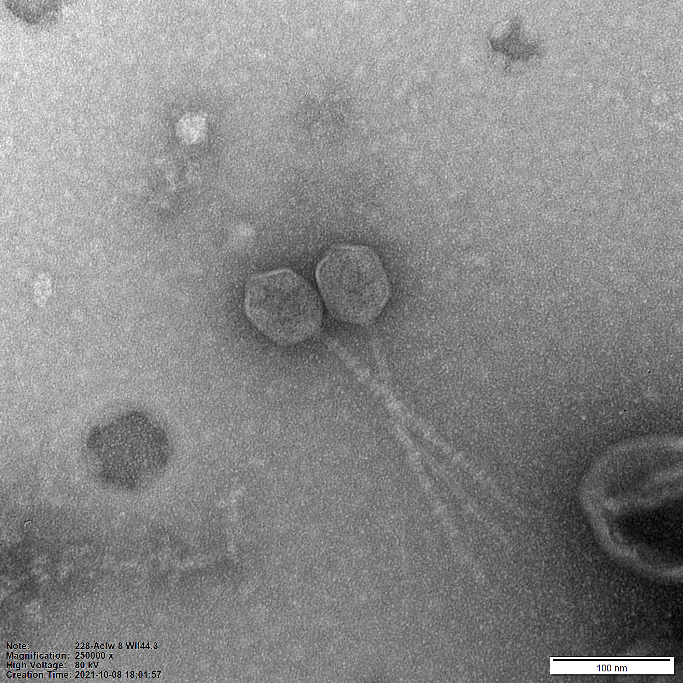

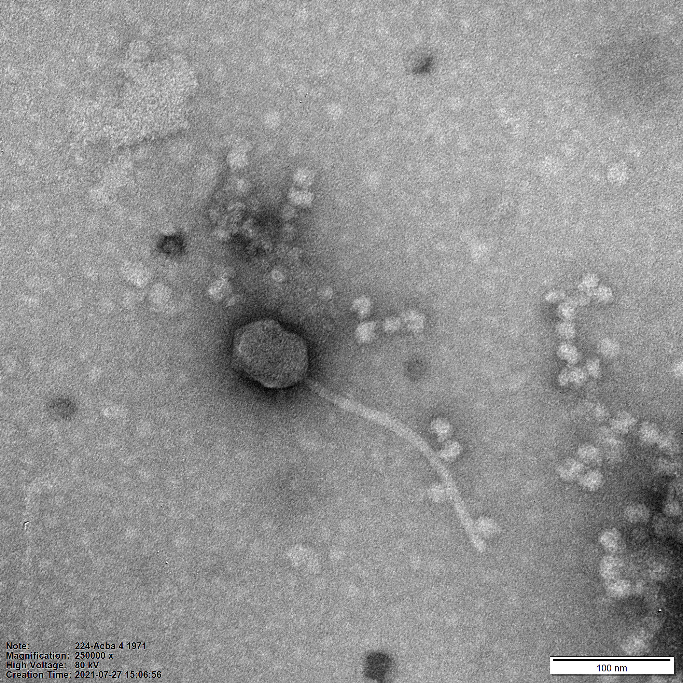


(B)

Acba_8

Acba_4

(A)


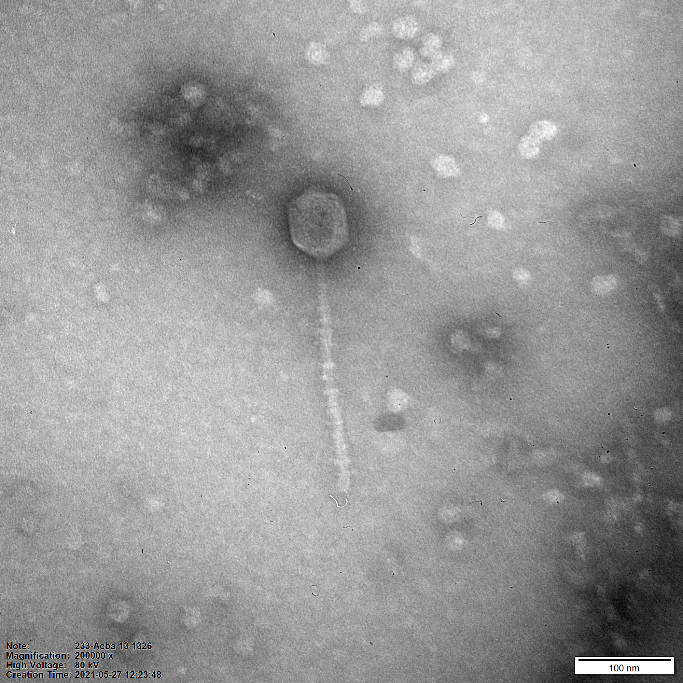

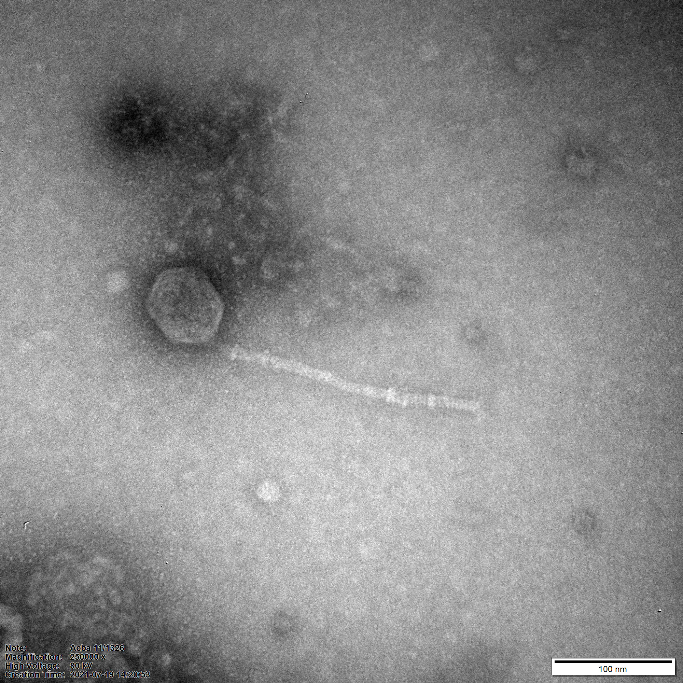


Acba_13

(D)

Acba_11

(C)


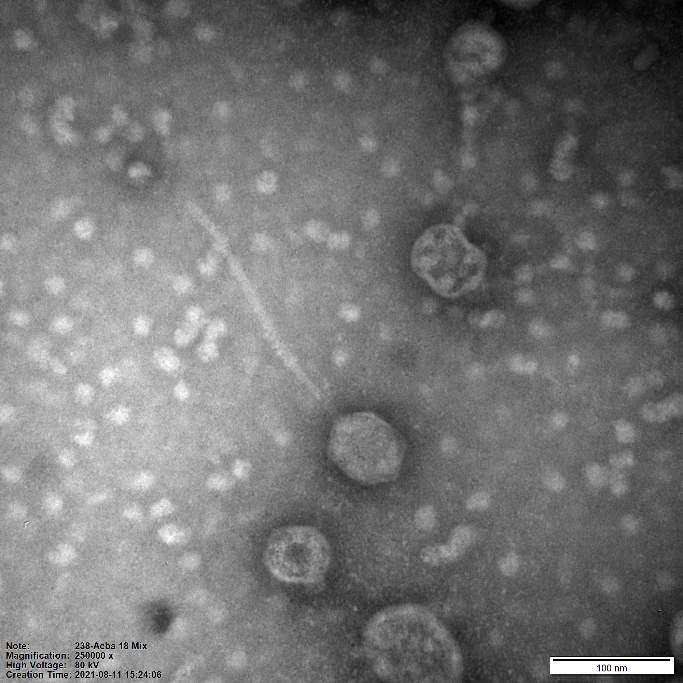

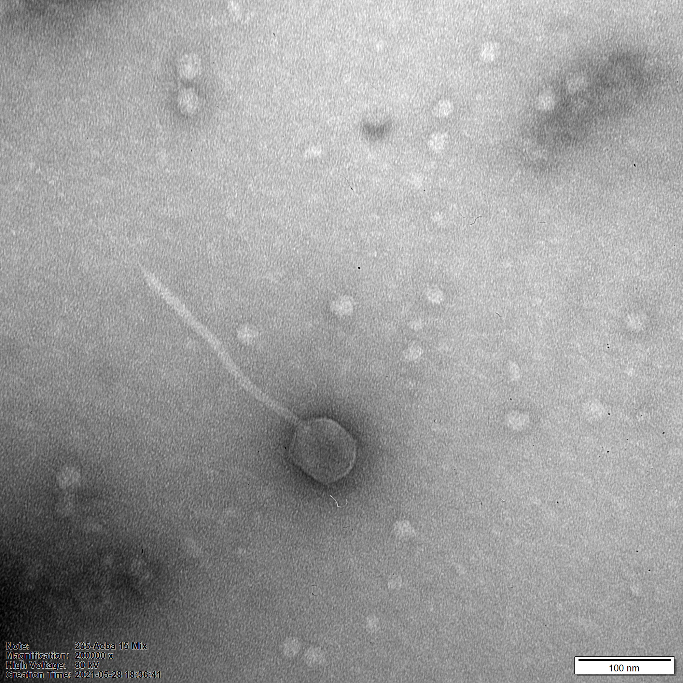

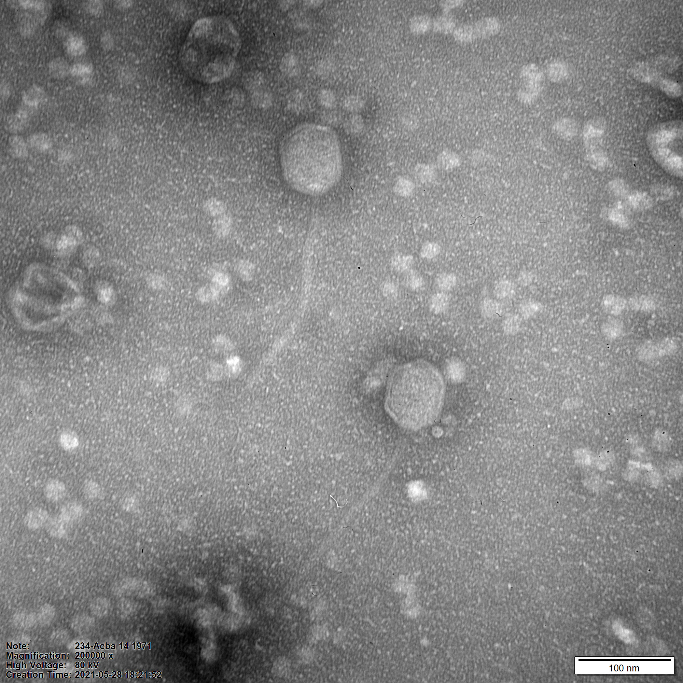


Acba_18

(G)

Acba_15

(F)

Acba_14

(E)

**Figure S2.** Electron micrographs from the TEM (Kodak/Carestream Electron Microscope) showing the morphology of selected bacteriophages. Panel (**A**): Acba_4, Panel (**B**): Acba_8, Panel (**C**): Acba_11, Panel (**D**): Acba_13, Panel (**E**): Acba_14, Panel (**F**): Acba_15, Panel (**G**): Acba_18. No electron micrograph was done for the Acba_1 phage. The Acba_13, Acba_14, Acba_15 phages were photographed at a magnification of 200,000x, for the Acba_4, Acba_8, Acba_11 and Acba_18 phage, a magnification of 250,000×. The reference bar shown on each panel corresponds to 100 nm.
