# Supplementary figures and images for: Biological Properties of 12 Newly Isolated Acinetobacter baumannii-Specific Bacteriophages
Source: Viruses. 2023 Jan 13;15(1):231. doi: 10.3390/v15010231 (PMC9866556; doi:10.3390/v15010231)

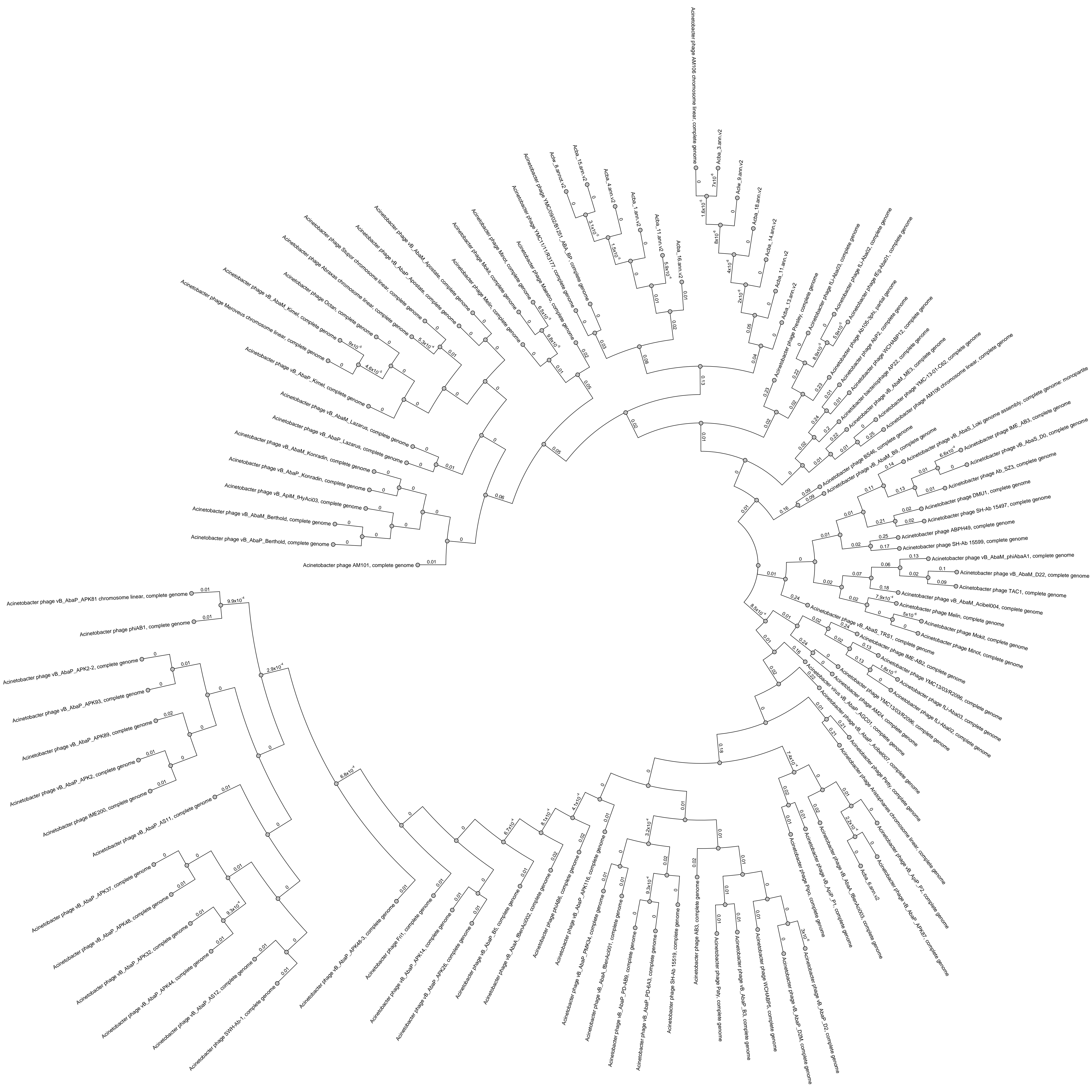

Supplement: Supplementary file 1 [file viruses-15-00231-s001.zip › data S1.pdf]
